# Fetal Supraventricular Tachycardia Complicated by Hydrops Fetalis: A Case Report of Complete Reversal With Transplacental Flecainide Therapy

**DOI:** 10.7759/cureus.117572

**Published:** 2026-10-07

**Authors:** Eleni Papaioannou, Aikaterini Martinopoulou, Ariadni Papadopoulou, Christos Kartsiounis, Magdalini Sterpi

**Affiliations:** 1 Department of Neonatology, Neonatal Intensive Care Unit, Hippokration General Hospital of Thessaloniki, Thessaloniki, GRC

**Keywords:** fetal arrhythmia, fetal supraventricular tachycardia, flecainide, neonatal arrhythmia, non-immune hydrops fetalis, transplacental therapy

## Abstract

Fetal supraventricular tachycardia (SVT) is the predominant sustained fetal tachyarrhythmia and, when persistent, can progress to tachycardia-induced myocardial dysfunction and nonimmune hydrops fetalis (NIHF), a condition associated with substantial perinatal morbidity and mortality. Although digoxin has historically served as first-line therapy, flecainide demonstrates superior transplacental bioavailability and higher cardioversion rates in hydropic fetuses, in whom placental edema may impair digoxin transfer. We report a case of fetal SVT complicated by hydrops fetalis at 30 weeks’ gestation that achieved complete intrauterine reversal following maternal flecainide therapy. A 31-year-old woman was referred after fetal echocardiography demonstrated SVT with a ventricular rate of approximately 300 beats per minute and 1:1 atrioventricular conduction, alongside fetal ascites and pericardial effusion consistent with NIHF. Oral flecainide was initiated and subsequently up-titrated because of persistent fetal tachycardia. Sinus rhythm was restored on treatment day 10, followed by complete sonographic resolution of hydrops by 32+6 weeks’ gestation. Labor was induced at 38 weeks, resulting in vaginal delivery of a clinically stable male infant. Postnatally, prophylactic propranolol was started, but recurrent SVT on days 4 and 13 of life required escalation to flecainide and adenosine cardioversion before discharge in sinus rhythm. This case illustrates that regression of hydrops, rather than restoration of sinus rhythm alone, represents a more clinically meaningful marker of fetal cardiovascular recovery and underscores the importance of timely transplacental antiarrhythmic therapy and structured postnatal surveillance, given the well-documented risk of arrhythmia recurrence in infants with a history of fetal SVT complicated by hydrops.

## Introduction

Fetal supraventricular tachycardia (SVT) is the most common sustained fetal tachyarrhythmia and represents the majority of clinically significant fetal rhythm disturbances encountered during pregnancy. Among fetal tachyarrhythmias, including sinus tachycardia, atrial flutter, and ventricular tachycardia, SVT accounts for the majority of cases requiring prenatal therapeutic intervention. The reported incidence varies across populations and study methodologies but has been estimated to range from approximately one in 1,000 to one in 25,000 pregnancies [1,2]. Persistent SVT may lead to progressive cardiovascular compromise due to sustained high ventricular rates, impaired diastolic filling, and increased systemic venous pressures. When untreated, these hemodynamic disturbances may culminate in tachycardia-induced myocardial dysfunction and nonimmune hydrops fetalis (NIHF), a severe fetal condition characterized by abnormal fluid accumulation in multiple fetal compartments and associated with significant perinatal morbidity and mortality. Hydrops develops in approximately 30-60% of fetuses with sustained SVT, and mortality rates in hydropic cases have been reported to approach 25-30%, particularly when tachycardia remains uncontrolled [2,3].

The pathophysiology of SVT-associated hydrops is primarily driven by prolonged tachycardia resulting in impaired ventricular relaxation, reduced ventricular filling time, and diminished cardiac output. As myocardial performance deteriorates, systemic venous pressures increase, promoting transudation of fluid into fetal serous cavities and interstitial tissues. This cascade leads to ascites, pleural and pericardial effusions, placental edema, and eventually generalized fetal edema. Contemporary fetal Doppler and M-mode echocardiographic studies have clarified this sequence of circulatory maladaptation and its progression to fetal cardiac failure [2,3]. Notably, tachycardia-induced hydrops represents a potentially reversible form of fetal heart failure, as the underlying cardiac structure is typically normal. Restoration of sinus rhythm may allow recovery of myocardial function, reduction of venous congestion, and gradual mobilization of accumulated fluid. Advances in fetal cardiology and transplacental pharmacotherapy have therefore substantially improved outcomes in pregnancies complicated by fetal tachyarrhythmias. Antiarrhythmic agents administered to the mother cross the placenta and achieve therapeutic fetal concentrations, enabling treatment of fetal arrhythmias without direct fetal intervention in most cases [2].

Digoxin has historically been used as first-line therapy for fetal SVT. However, accumulating evidence suggests that flecainide and sotalol may achieve higher cardioversion rates, particularly in hydropic fetuses, in whom placental edema may impair the transplacental transfer of digoxin and potentially reduce fetal drug exposure [2-5].

Despite advances in fetal therapy, the dynamic relationship between rhythm control and regression of hydrops remains a key determinant of fetal stabilization and perinatal outcome, and understanding this relationship is critical for guiding therapeutic decisions in hydropic fetuses. We report a case of fetal SVT complicated by NIHF at 30 weeks’ gestation with complete intrauterine reversal following maternal flecainide therapy. This case illustrates the pathophysiological reversibility of tachycardia-induced hydrops and highlights the importance of timely transplacental antiarrhythmic treatment in optimizing fetal stabilization and achieving favorable neonatal outcomes.

## Case presentation

A 31-year-old gravida 2, para 1 (G2P1) woman with an otherwise uncomplicated pregnancy was referred at 30 weeks’ gestation after persistent fetal tachycardia was detected during routine antenatal assessment. She had no significant medical history, was not receiving any medications, and reported no family history of cardiac disease or arrhythmias.

Fetal echocardiography demonstrated sustained SVT with a ventricular rate of approximately 300 beats per minute and 1:1 atrioventricular conduction. Structural cardiac anatomy was normal. Ultrasound examination revealed fetal ascites and a moderate pericardial effusion, fulfilling sonographic criteria for NIHF (Figure 1).

**Figure 1:**
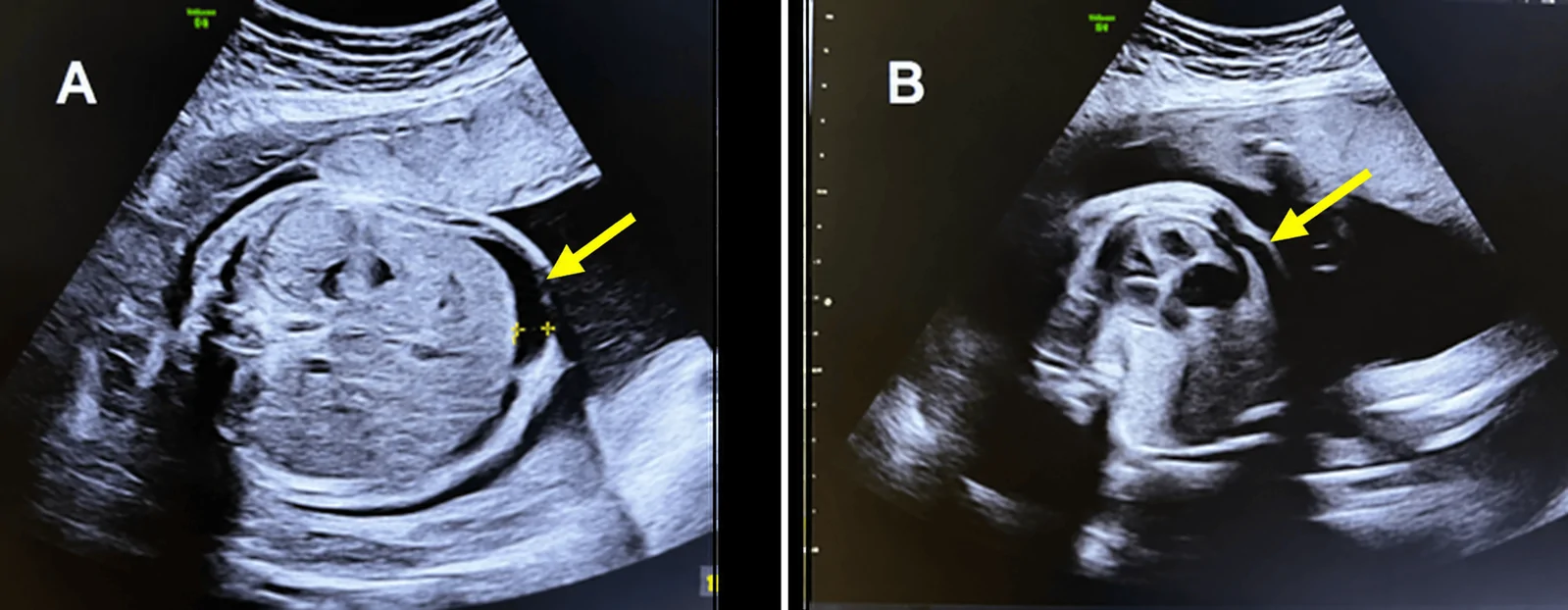
Prenatal ultrasonography at diagnosis of NIHF. Prenatal ultrasound obtained at 30 weeks’ gestation demonstrating the principal sonographic features of NIHF in a fetus with sustained SVT. (A) Fetal ascites (arrow). (B) Moderate pericardial effusion (arrow). NIHF, nonimmune hydrops fetalis; SVT, supraventricular tachycardia

Maternal evaluation was undertaken to identify potential secondary causes of fetal tachycardia. Laboratory investigations, including thyroid function tests and infectious disease screening, were unremarkable.

Given the presence of sustained SVT complicated by hydrops fetalis, transplacental antiarrhythmic therapy with oral flecainide was initiated at a dose of 100 mg three times daily. Management was undertaken on an inpatient basis with clinical surveillance and serial maternal electrocardiography reviewed by a cardiologist. Because persistent fetal tachycardia was observed, the flecainide dose was increased to 100 mg four times daily after seven days of treatment. Maternal serum flecainide concentrations were not measured because therapeutic drug monitoring was not available at our institution. No clinically significant conduction or repolarization abnormalities or other maternal adverse effects were reported throughout therapy.

Restoration of fetal sinus rhythm was achieved on treatment day 10. Serial fetal ultrasonographic examinations demonstrated progressive regression of hydrops following rhythm conversion, with gradual resolution of fetal ascites and pericardial effusion over the subsequent days. By 32+6 weeks’ gestation, follow-up fetal ultrasonography demonstrated complete resolution of NIHF (Figure 2). Fetal cardiac function improved, and no further fetal deterioration was observed. The antenatal clinical course is summarized in Table 1. Maternal flecainide therapy was continued for the remainder of the pregnancy. At 38 weeks’ gestation, labor was induced, resulting in vaginal delivery of a male infant without perinatal complications. Birth weight was 2290 g (<3rd percentile for gestational age), and Apgar scores were 8 and 9 at one and five minutes, respectively. Fetal growth restriction had not been diagnosed antenatally; antenatal surveillance showed no abnormal umbilical artery Doppler findings, abnormal cerebroplacental ratio, or other evidence of placental insufficiency. The infant was therefore classified as small for gestational age at birth.

**Figure 2:**
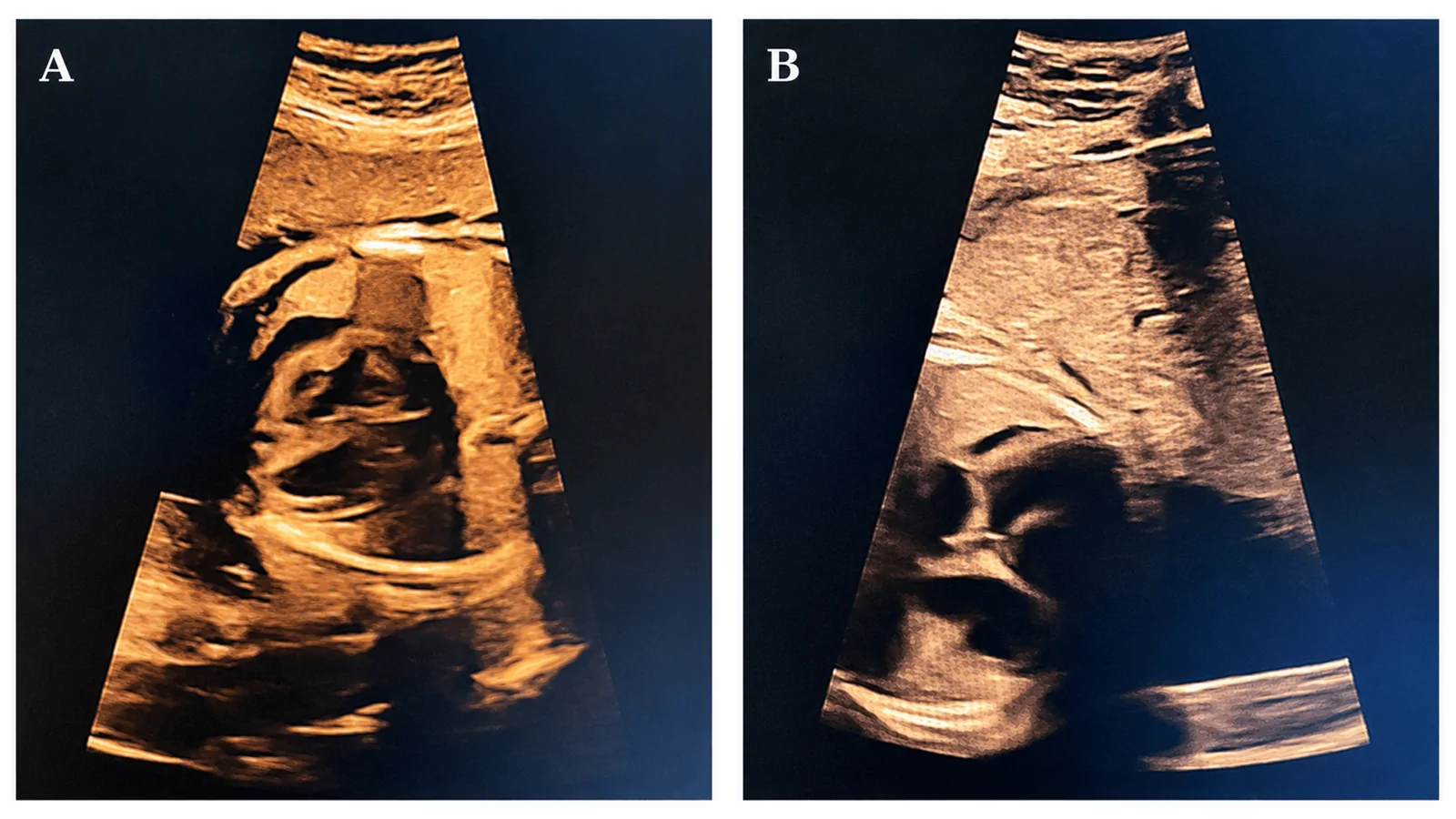
Serial fetal ultrasonography following transplacental flecainide therapy. Serial follow-up fetal ultrasonography after restoration of sinus rhythm with transplacental flecainide therapy demonstrating regression of hydrops fetalis. (A) Complete resolution of the pericardial effusion. (B) Resolution of fetal ascites.

**Table 1:** Clinical timeline illustrating the antenatal management of fetal SVT and the subsequent resolution of NIHF. NIHF, nonimmune hydrops fetalis; SVT, supraventricular tachycardia

Gestational age	Treatment day	Clinical event
30+0 weeks	Day 0	Sustained fetal SVT (~300 bpm) with NIHF (fetal ascites and moderate pericardial effusion); transplacental flecainide initiated (100 mg three times daily)
31+0 weeks	Day 7	Flecainide increased to 100 mg four times daily due to persistent fetal tachycardia
31+3 weeks	Day 10	Restoration of fetal sinus rhythm
32+0 weeks	Day 14	Progressive regression of fetal ascites and pericardial effusion
32+6 weeks	Day 20	Complete resolution of NIHF
38+0 weeks	-	Vaginal delivery of a male infant

Neonatal course

Following delivery, the neonate was admitted to the neonatal unit for continuous cardiorespiratory monitoring. Clinical examination demonstrated no evidence of residual hydrops fetalis. Initial electrocardiography showed normal sinus rhythm, while postnatal echocardiography confirmed structurally normal cardiac anatomy and normal ventricular function (Figure 3). Oral propranolol therapy was initiated on the first day of life as prophylactic antiarrhythmic therapy.

**Figure 3:**
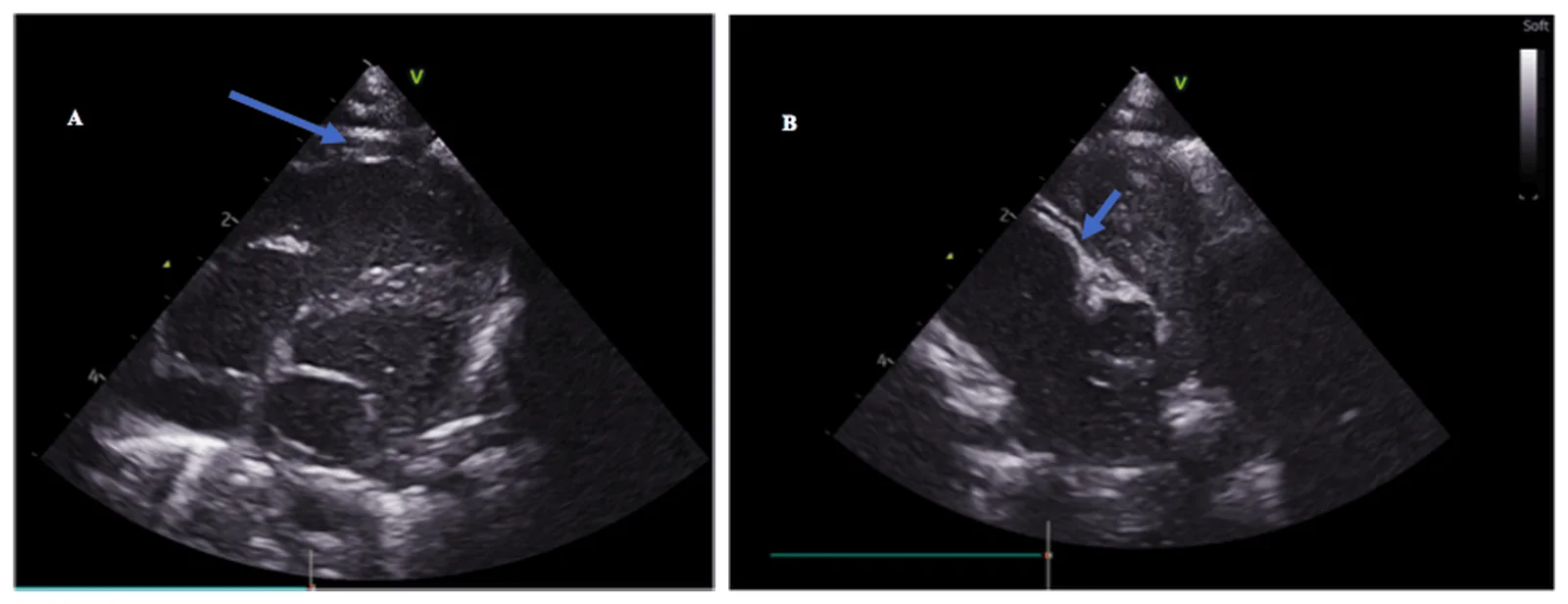
Postnatal transthoracic echocardiography. (A) Apical four-chamber view demonstrating structurally normal cardiac anatomy. (B) Follow-up transthoracic echocardiographic view demonstrating normal biventricular systolic function with complete resolution of the pericardial effusion.

On day 4 of life, two brief episodes of SVT were documented. Both episodes were self-terminating and hemodynamically well tolerated. The propranolol dose was subsequently increased. On day 13 of life, a further episode of SVT occurred and required pharmacologic cardioversion with adenosine. Following conversion to sinus rhythm, electrocardiographic assessment by a pediatric cardiologist showed no evidence of ventricular pre-excitation or a Wolff-Parkinson-White pattern. Flecainide was subsequently added to the antiarrhythmic regimen.

After optimization of medical therapy, the infant remained hemodynamically stable in sustained sinus rhythm. He was discharged home on day 20 of life in good clinical condition with ongoing follow-up in the pediatric cardiology clinic.

## Discussion

Hydrops regression as a marker of cardiovascular recovery and prognosis

Fetal SVT complicated by hydrops fetalis represents a form of tachycardia-induced fetal cardiac failure that may be reversible once adequate rhythm control is achieved. Sustained tachycardia impairs ventricular filling, reduces cardiac output, and increases systemic venous pressure, resulting in fluid accumulation within fetal serous cavities and soft tissues. Consequently, hydrops serves not only as a marker of disease severity but also as a dynamic indicator of therapeutic response. Emerging evidence suggests that the clinically relevant milestone is not simply restoration of sinus rhythm, but regression of hydrops following rhythm control, reflecting recovery of myocardial performance and reversal of fetal cardiovascular decompensation [1,3,6,7].

Several studies have demonstrated the close relationship between hydrops regression and favorable perinatal outcomes. In the 20-year series reported by Tunca Sahin et al., hydropic fetuses treated with flecainide- and digoxin-based regimens achieved high rates of rhythm control, with hydrops resolving in the majority of survivors. Similarly, Krapp et al. observed that hydrops regression generally followed successful cardioversion, although the interval to complete resolution varied considerably among cases. These observations suggest that restoration of sinus rhythm initiates, but does not immediately complete, the process of fetal cardiovascular recovery [7,8].

Hydrops regression appears to be a more clinically meaningful marker of fetal cardiovascular recovery than rhythm conversion alone, as fetal demise may occur despite successful cardioversion when hydrops fails to resolve. Supporting this concept, Broom et al. reported two hydropic fetuses who achieved successful rhythm control but subsequently died because hydrops persisted, indicating that restoration of sinus rhythm does not invariably translate into recovery of cardiac function or reversal of end-organ compromise. Consequently, regression of hydrops may represent a more robust indicator of myocardial recovery and improved prognosis than rhythm normalization alone [6,7].

In the present case, sinus rhythm was restored within 10 days of treatment initiation, whereas complete regression of hydrops occurred 10 days later. This temporal sequence is consistent with previous reports and suggests that recovery of myocardial function and normalization of venous pressures continue after successful cardioversion. The lag between rhythm conversion and hydrops resolution highlights that restoration of sinus rhythm alone does not immediately reverse the hemodynamic consequences of prolonged tachycardia-induced cardiac failure. Rather, regression of hydrops appears to represent the final stage of fetal cardiovascular recovery. In our case, complete resolution of hydrops was followed by continuation of pregnancy to term and an excellent neonatal outcome, further supporting the prognostic significance of hydrops regression as a marker of successful fetal therapy.

Time to hydrops regression

Although rhythm conversion may occur rapidly after initiation of antiarrhythmic therapy, regression of hydrops typically follows a more gradual course. The literature consistently demonstrates that hydrops resolution lags behind restoration of sinus rhythm, highlighting that cardioversion and true cardiovascular recovery are related but distinct therapeutic milestones.

As shown in Table 2, published studies consistently demonstrate that hydrops regression occurs after restoration of sinus rhythm and is associated with improved fetal and neonatal outcomes. Although the timing of hydrops resolution varies considerably across reports, regression generally lags behind cardioversion by several days to weeks, reflecting the time required for recovery of cardiovascular function and reabsorption of accumulated fluid.

**Table 2:** Published studies evaluating the temporal relationship between rhythm control, hydrops regression, and neonatal outcomes in fetal SVT complicated by hydrops fetalis. SVT, supraventricular tachycardia

Study	Study design	Hydropic fetuses	Cardioversion rate	Time to rhythm control	Time to complete hydrops regression	Neonatal outcome
Cui et al. [1]	Case report	1	SVT resolved after digoxin + sotalol	~3-4 days	Progressive regression	Delivered at 36+5 weeks by cesarean for recurrent SVT, abnormal ductus venosus Doppler, and oligohydramnios; neonate stable
Andonotopo et al. [3]	Systematic review/meta-analysis	Multiple cohorts	Digoxin ~30-40%; flecainide ~70-80% in hydropic fetuses	Variable	Hydrops regression following cardioversion	Survival strongly associated with hydrops resolution
Broom et al. [6]	Single-center cohort	23/69	81.5% with combination therapy	Flecainide ~3 days; digoxin ~5 days	Not reported	Overall mortality 4.3%; favorable outcome when hydrops resolved
Tunca Sahin et al. [7]	20-year retrospective cohort	20/20	90% tachycardia response; 85% sinus rhythm	Median 1.5 days (12 hours-13 days)	Median 12 days (3-21)	Excellent survival; one postnatal death related to prematurity
Krapp et al. [8]	Prospective series	15/20	95% (19/20)	Median 4 days (1-12)	Median 8 days after cardioversion (2-33)	All fetuses survived; occasional postnatal SVT recurrence
Narayanan et al. [9]	Case report	1	Sinus rhythm within 24 hours	~1 day	Ascites resolved by day 4	Neonate stable; no persistent arrhythmia
Munoz et al. [10]	Case report	1	Cardioversion after direct fetal therapy	Rapid	Hydrops persisted	Delivery required due to maternal mirror syndrome
Agarwal et al. [11]	Case report	1	Refractory SVT	Delayed	Persistent hydrops	High-risk course requiring intervention
Minisha et al. [12]	Case report	1	Refractory tachycardia	Delayed	Progressive hydrops	Poorer outcome compared with responsive cases
Present case	Case report	1	Sinus rhythm restored within ~10 days	~10 days	Complete hydrops regression 10 days after rhythm control	Favorable neonatal outcome

In the series reported by Tunca Sahin et al., rhythm control was generally achieved early, whereas hydrops resolved after a median of 12 days (range, 3-21 days). Similarly, Krapp et al. observed hydrops resolution at a median of 8 days (range, 2-33 days) after cardioversion among early responders and 15 days (range, 5-31 days) among late responders, all of whom were hydropic. When measured from treatment initiation, hydrops resolution occurred after a median of 11 days (range, 5-38 days) in early responders and 27 days (range, 18-40 days) in late responders [7,8].

Our case closely mirrored this temporal pattern. Restoration of sinus rhythm was achieved on treatment day 10, whereas complete resolution of hydrops occurred 10 days later. This sequence supports previous observations that recovery of fetal cardiovascular function and mobilization of accumulated fluid continue after successful cardioversion and may require prolonged surveillance despite normalization of cardiac rhythm.

Case reports provide additional illustration of this sequence. In the report by Narayanan et al., combined digoxin and flecainide therapy restored sinus rhythm within 24 hours, followed by rapid reduction of ascites and effusions, with complete resolution of ascites by day 4 [9]. Similar observations have been described in other reports, supporting the concept that successful rhythm control is frequently followed by progressive regression of hydrops under continued fetal surveillance rather than necessitating immediate delivery [13,14].

Compared with these reports, our case demonstrated a somewhat slower but equally favorable pattern. Sinus rhythm was restored on treatment day 10, followed by progressive hydrops regression, with complete resolution 10 days later. Nevertheless, the overall recovery trajectory remained well within the range reported for successfully treated hydropic fetuses and ultimately allowed continuation of pregnancy to term and delivery of a clinically stable neonate.

Comparative efficacy of antiarrhythmic therapy in hydropic SVT

Drug efficacy in fetal SVT differs substantially according to the presence or absence of hydrops (Table 3). Contemporary reviews and meta-analyses have demonstrated that digoxin, although historically considered first-line therapy, is considerably less effective in hydropic fetuses than in non-hydropic cases. According to comparative analyses summarized by Andonotopo et al. and Tsokkou et al., digoxin achieves approximately 55-70% efficacy in non-hydropic fetuses but only 30-40% in hydropic fetuses. In contrast, flecainide maintains high efficacy in both settings, with reported success rates of approximately 80-90% in non-hydropic fetuses and 70-80% in hydropic fetuses. Sotalol demonstrates intermediate efficacy, whereas amiodarone is generally reserved for refractory cases because of its less favorable safety profile and limited transplacental transfer [2,3].

**Table 3:** Comparative efficacy, placental transfer, and clinical considerations of antiarrhythmic drugs used for fetal SVT. Original table created by the authors based on data extracted and synthesized from the cited literature. SVT, supraventricular tachycardia

Drug	Pharmacologic class/mechanism	Efficacy in non-hydropic SVT	Efficacy in hydropic SVT	Placental transfer	Clinical considerations
Digoxin	Cardiac glycoside; AV nodal blockade with positive inotropic effect	≈55-70% [2]	≈30-40% [2]	Reduced in hydropic pregnancies; fetal-to-maternal ratio ≈0.8-1 [15]	Traditionally first-line therapy; often combined with flecainide to improve efficacy [2,15]
Flecainide	Class Ic sodium channel blocker	≈80-90% [2]	≈70-80% [2]	High transplacental transfer; fetal-to-maternal ratio ≈0.8-0.95 [15]	Highly effective in hydropic SVT; rapid cardioversion and strong transplacental bioavailability [2,15]
Sotalol	Class III antiarrhythmic with non-selective beta-blocking activity	≈65-75% [2]	≈60-70% [2]	High but variable transplacental transfer; fetal-to-maternal ratio ≈0.7-2.9 [15]	Particularly useful for long-VA SVT and atrial flutter; requires QT monitoring [2,15]
Amiodarone	Class III multi-channel antiarrhythmic	≈50-60% [2]	≈50-60% (limited data) [2]	Poor transplacental transfer; fetal-to-maternal ratio ≈0.1-0.28 [15]	Reserved for refractory tachyarrhythmias due to potential maternal and fetal adverse effects [2,15]

These differences are clinically important because successful rhythm control is a prerequisite for subsequent hydrops regression. Meta-analytic evidence further supports the superiority of flecainide over digoxin for termination of fetal SVT, particularly in hydropic fetuses, in whom digoxin was associated with a significantly lower tachycardia termination rate than flecainide (OR 0.412; 95% CI 0.268-0.632) [2].

The favorable response observed in our patient is consistent with these findings. Despite presenting with established NIHF, restoration of sinus rhythm and subsequent hydrops regression were achieved following flecainide therapy, supporting the growing body of evidence that flecainide-containing regimens represent an effective therapeutic strategy for hydropic fetal SVT.

Clinical implications and integration with our case

An important observation emerging from the literature is that restoration of sinus rhythm alone does not guarantee a favorable outcome if hydrops persists. In the cohort reported by Broom et al., all fetal deaths occurred in hydropic fetuses, and notably, two deaths occurred despite successful rhythm conversion because hydrops failed to resolve. Similarly, reports of refractory fetal SVT describe persistent hydrops leading to urgent delivery, invasive fetal intervention, maternal mirror syndrome, or fetal demise [6,10-12]. These observations emphasize that successful cardioversion should be regarded as an intermediate therapeutic milestone, whereas regression of hydrops more accurately reflects true cardiovascular recovery.

Our case followed the opposite and more favorable trajectory. Restoration of sinus rhythm was achieved within 10 days of treatment initiation and was subsequently followed by progressive resolution of ascites and pericardial effusion, culminating in complete regression of NIHF. This recovery pattern permitted continuation of pregnancy to term and resulted in a favorable neonatal outcome. The sequence observed in our patient closely resembles the “successful reversal” phenotype reported in the largest published series, in which rhythm control is followed by hemodynamic recovery, hydrops regression, and survival [6-9].

The neonatal course also highlights an important aspect of long-term management. Despite complete prenatal rhythm control and hydrops resolution, recurrent SVT episodes occurred during the early postnatal period. This finding is consistent with published experience demonstrating that postnatal arrhythmia recurrence may occur despite successful intrauterine treatment and likely reflects persistence of the underlying electrophysiologic substrate rather than failure of prenatal therapy [1,2].

Contemporary pediatric cardiology guidance similarly emphasizes the importance of postnatal rhythm surveillance in infants with a history of fetal tachyarrhythmias. According to the 2024 American Heart Association scientific statement on fetal and neonatal arrhythmia management, at least half of infants diagnosed with fetal SVT experience postnatal recurrence, typically within the first two to three days of life; reported predictors of recurrence include fetal hydrops, delayed or absent prenatal cardioversion, need for multidrug prenatal therapy, and a long ventriculoatrial interval [15]. Consequently, close monitoring and, in selected cases, prophylactic antiarrhythmic therapy are recommended to reduce the risk of clinically significant recurrence. The presence of antenatal hydrops in our patient is therefore consistent with an elevated a priori risk of postnatal recurrence. In our patient, propranolol was initiated shortly after birth; however, recurrent SVT was nonetheless observed on day 4 of life and ultimately required escalation of therapy with flecainide, illustrating the need for continued vigilance even after successful prenatal treatment.

Taken together, the available evidence and our experience support a central clinical concept: in fetal SVT complicated by hydrops fetalis, regression of hydrops should be considered the most clinically meaningful marker of successful fetal rescue. While restoration of sinus rhythm remains essential, complete hydrops regression more accurately reflects recovery of cardiovascular function and is consistently associated with improved perinatal and neonatal outcomes.

## Conclusions

Fetal SVT complicated by hydrops fetalis represents a form of tachycardia-induced fetal cardiac failure that is potentially reversible with timely intrauterine therapy. Restoration of sinus rhythm through transplacental antiarrhythmic treatment may allow recovery of myocardial function and gradual regression of hydrops, thereby stabilizing fetal hemodynamics and permitting continuation of pregnancy. The present case illustrates the characteristic sequence described in the literature: successful cardioversion, progressive resolution of hydrops, and favorable neonatal outcome. Importantly, hydrops regression may be the most clinically meaningful marker of cardiovascular recovery in hydropic fetal SVT. Despite successful prenatal rhythm control, neonatal recurrence remains common, underscoring the importance of structured postnatal monitoring and individualized antiarrhythmic management. Early diagnosis, multidisciplinary fetal-neonatal collaboration, and timely pharmacologic intervention remain essential for optimizing both fetal stabilization and neonatal prognosis.
